# Long noncoding RNA PP7080 promotes hepatocellular carcinoma development by sponging mir-601 and targeting SIRT1

**DOI:** 10.1080/21655979.2021.1920323

**Published:** 2021-05-06

**Authors:** Weifang Song, Zhang Wenhui, Yao Ruiqiang, Xinli Hu, Ting Shi, Meijiao Wang, Haijuan Zhang

**Affiliations:** a Department of Pathophysiology, Fenyang College of Shanxi Medical University, Fenyang, China; b Department of Clinical Medicine, Fenyang College of Shanxi Medical University, Fenyang, China

**Keywords:** HCC, lncRNA pp7080, miR-601, sirt1

## Abstract

Hepatocellular carcinoma (HCC) is the most common primary liver malignancy in adults, ranking the second leading cause of cancer-related death. To date, the underlying mechanisms of HCC pathogenesis are still unclear. Recently, more and more studies have reported that long noncoding RNAs (lncRNAs) are involved in the occurrence and development of HCC. This study aims to investigate the expressions, clinical significance and roles of lncRNA PP7080 in HCC. We analyzed the transcriptome data of HCC cancer tissue (*n* = 369) and normal tissue (*n* = 50) in the TCGA database. We used the qRT-PCR method to detect the expression levels of lncRNA PP7080 in 40 pairs of HCC and adjacent tissues. The survival curve was drawn by KM-plotter. The changes of migration, invasion and proliferation of HCC cells were detected by transwall, CCK8 and colony forming assays, respectively. For the interaction between genes, we performed the luciferase activity assay to analyze. The expression of lncRNA PP7080 and miR-601 in cancer tissues of 40 cancer patients was analyzed by Pearson correlation. LncRNA PP7080 was highly expressed in HCC and predicted a poor prognosis. Luciferase activity assay identified lncRNA PP7080 as a molecular sponge for miR-601 in HCC cells. LncRNA PP7080 promoted HCC cells proliferation, migration and invasion by miR-601/SIRT1 signal axis. These results revealed lncRNA PP7080 effect in regulating miR-601/SIRT1 signal axis in the progression of HCC, indicating the important role of miR-601 in HCC pathogenesis.

## Graphical abstract

**Figure:**
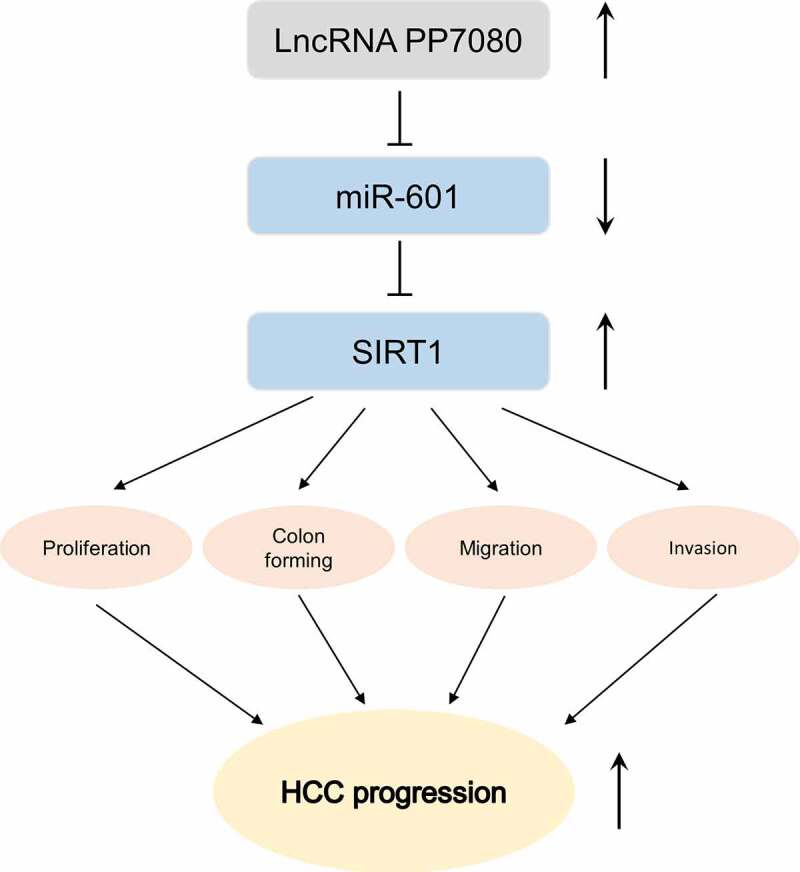

## Introduction

Hepatocellular carcinoma (HCC) represents the third largest cause of cancer mortality worldwide and the most common type of malignant tumor in humans[1], [2]. Only a small percentage of HCC patients undergo radical surgery, and people who are applicable for radiotherapy have a high risk of recurrence. Although the prevention, diagnosis and intervention of HCC have made progress in recent years, its treatment is still not satisfactory [2]. Hence, it is urgent to clarify the underlying mechanism of HCC progression, and to search novel potential HCC therapeutic targets. In recent years, increasingly evidences have revealed that noncoding RNA plays a regulatory role in a variety of tumors including HCC [3,4], suggesting the noncoding RNA crucial role in HCC diagnosis and intervention.

MicroRNAs (miRNAs) are a large group of small noncoding RNA that inhibits or induces the translation or degradation of target mRNA through interacting with the 3ʹUTR of target mRNA [5–7]. miRNA is a key regulator in many biological processes, including cell proliferation, apoptosis, migration, differentiation and cell cycle [8]. Dysregulation of miRNAs plays a key role in several human tumorigenesis, progression and metastasis. Meanwhile, miRNAs can act as an oncogene or antioncogene. A recent study proved that miRNAs may be used as biomarkers for the diagnosis or prognosis of breast cancer [9]. Therefore, miRNAs might also be potential diagnostic biomarkers and therapeutic targets for HCC. Previous studies have indicated that plasma miR-601 level was significantly downregulated in colorectal cancer [10]. MiR-601 can affect multiple signaling pathways in lung cancer cell A549 [11]. However, the biological role of mir-601 in HCC is largely unclear.

In recent years, plenty of attention was paid for long noncoding RNAs (lncRNAs) in cancer research [12]. Emerging evidences indicated various lncRNAs are dysregulation and involved in the progression and pathogenesis of several cancers, including HCC [12,13]. Recently, lncRNA PP7080 has been suggested as a promising biomarker for the diagnosis and prognosis of colon adenocarcinoma (COAD) [14]. As a newly identified lncRNA biomarker for COAD, the biological functions of lncRNA PP7080 remain unknown. Meanwhile, whether lncRNA PP7080 can be also served as a candidate biomarker for diagnosis and prognosis of other cancers needs to be further studied. Previous study revealed that SIRT1 affected cell senescence through maintaining normal mitochondrial function under oxidative stress, regulating metabolism (calorie consumption, fat storage, etc.), inhibiting inflammation and inhibiting cell apoptosis [15]. Additionally, recent studies found that SIRT1 may be a potential therapeutic target in HCC [16]. However, the underlying regulation mechanism of SIRT1 in HCC progression is still unclear.

In this study, we aimed to evaluate the expression levels of lncRNA PP7080 in HCC and confirmed the relationship between lncRNA PP7080 and the prognosis of HCC patients, and we also investigate its effects and underlying mechanism of lncRNA PP7080 in promoting HCC progression. Our study showed that lncRNA PP7080 as a molecular sponge for targeting miR-601 and regulating SIRT1 signal to promote HCC cells proliferation, migration and invasion. Our findings indicate the crucial role of lncRNA PP7080 in HCC pathogenesis.

## Materials and methods

### Reagents

The information of the main reagents involved in this study is as follows: DMEM medium (SH30022.01B, GE™ Hyclone), FBS (SH30087.01, GE™ Hyclone), penicillin (SH30010, GE™ Hyclone) and PBS (SH30256.01B, GE™ Hyclone); Lipofectamine™ RNAiMAX (13,778–075, Invitrogen, USA); Cell Counting Kit-8 (CCK-8, Key GEN Bio TECH, China).

### TCGA database analysis

The expression level of PP7080 in HCC and adjacent tissue was analyzed by GEPIA software (http://gepia.cancer-pku.cn/) based on the TCGA public database. The results are shown using box plots.

### Cell culture and vector transfection

HCC cells Huh7 and HepG2 were obtained from DSMZ (Braunschweig, Germany), maintained in complete cell culture medium (DMEM medium, 10% FBS, 100 mg/ml streptomycin and 100 U/ml penicillin) and cultured in humidified atmosphere at 37°C with 5% CO_2_. PP7080 shRNA (#1 forward: CAGGAGGAGTTCTTAAAGAG and #2 forward:TTTGGGATTCAGTGGTTATTC), miR-601inh or SIRT1 overexpression plasmid was transfected into HEK293T cell by Lipofectamine® 3000.

### Stable cell line generation

For PP7080 knock-down stable cell line generation: sh-PP7080 co-transfected with lentivirus packaging vectors into HEK293T cells with lipo2000; after 12 h, discard the old medium and replace it with fresh medium, and continue to culture for 36 h; then collect the supernatant medium, filter with a 0.45 μm filter to obtain the virus liquid. Before infection, the indicated cell line was seeded in 24-well plate for 12 h, then the collected virus was added with 8 μg/ml polybrene; 48 h later, the cell culture medium was replaced with a complete medium containing 1 μg/ml puromycin for selection. After around 72 h later, the selected cells were prepared for subsequent experiments.

### Quantitative reverse transcription PCR

Total mRNA was isolated from Huh7 and HepG2 using the Trizol reagent (#15596018, Life Technologies) and reversed transcribed into cDNA using RNA reverse transcription kit (#205313, abm). The 2-ΔΔCt method was used to calculate the PP7080 (F: ACCTCCCGAGCGCCAGGACT, R: GATCTGCAGTTCAAAGACCTGGC), SirT1 (F: TAGACACGCTGGAACAGGTTGC, R: CTCCTCGTACAGCTTCACAGTC), mir-601 (F: GGTCTAGGATTGTTGGAG, R: GAACATGTCTGCGTATCTC) expression and normalized by GAPDH (F: GTGGACCTGACCTGCCGTCT, R: GGAGGAGTGGGTGTCGCTGT).

### Western blot

In order to detect the expression level of SIRT1 protein in cells, we used the western blot method to detect the proteins extracted from Huh7 and HepG2. The whole-cell lysate was extracted with lysis buffer. The concentration of the extracted protein was measured by Bradford method. Soluble proteins (30–40 μg) were separated by SDS-polyacrylamide gel electrophoresis and transferred to polyvinylidene fluoride (PVDF) membrane (Millipore, Billerica, MA, USA). The PVDF membrane was blocked with 5% BSA for 1 h and incubated overnight with mouse-anti-human SirT1 primary antibody (8469S; ell Signaling Technology, USA). GAPDH was used as endogenous controls. The membrane was washed with TBST three times, 5 min each time, and incubated with secondary antibodies for 1 h at room temperature. After washing with TBST three times, 5 min each time, and then the PVDF film was developed after adding the developer. The primary antibody used in this study was diluted into 5% BSA at a ratio of 1:1000.

### Cell proliferation test

In the cell proliferation test, 1 × 10^3^ cells in the logarithmic growth phase required for the corresponding experiment were seeded in each well of a 96-well plate and cultured overnight at 37°C and 5% CO_2_. Then, we added 10 µL of CCK-8 to each well and incubated at 37°C for 2–4 h. After the culture was terminated, the absorbance at 450 nm was measured with a microplate reader, and the experiment was repeated three times.

### Colony formation assay

At 48 h after transfection, cells were digested and seeded at a density of 1 * 10^3^ cells per well in a six-well plate. Cells were routine cultured in DMEM medium containing 10% FBS, streptomycin and penicillin in humidified atmosphere at 37°C with 5% CO_2_ for 15 days. The medium was discarded and fixed with 4% paraformaldehyde for 15 min when visible clones appeared in the Petri dish. After being treated with 0.1% crystal violet dye for 10 min and washed several times with PBS, the number of clones was counted.

### Transwell assay

Cells in the logarithmic growth stage were starved for 24 h, and digested the cells the next day, centrifugated and resuspended, with a final concentration of 2 × 10^5^/ml. Then the procedure was performed according to the method and steps described in the section ‘Induction and detection of human mesenchymal stem cell migration in the 48-well reusable transwell assay’ [17]. The number of transmembrane cells was counted in five randomly selected fields (100×) using an inverted microscope.

### Luciferase reporter gene technique

The mutation sequence of PP7080 binding sites targeting the adsorption of Mir-601 was designed. The above binding sites and the mutated sequence were constructed into the luciferin reporter gene and transfected into HepG2, respectively. Then miR-601 was overexpressed to detect the expression of luciferin. Dual Luciferase Gene Reporter Assay was previously described in the ‘Dual Luciferase Gene Reporter Assays to Study miRNA Function’[18].

### Statistical analysis

The expression protein in cancer tissues of HCC patients was analyzed by Pearson correlation analysis. All experiments in this study were repeated at least three times, and all data are expressed as mean ± SD. The differences between the two independent experimental groups were tested by two-tailed Student’s *t*-tests. One-way analysis of variance followed by Tukey's multiple-comparison test was applied for data comparison between different groups in the current study. Differences were considered significant if *p* < 0.05: *p < 0.05; **p < 0.01; ***p < 0.001.

## Results

Recently, emerging evidences indicated that lncRNA plays an important regulatory role in the progression of HCC. A previous study suggested that lncRNA PP7080 acted as a biomarker of colorectal cancer. Herein, we suppose that lncRNA PP7080 is associated with the pathogenesis of HCC. We first confirmed that the level of lncRNA PP7080 exhibits high expression in HCC and relates with the prognosis of HCC patients. Then our study proved that lncRNA PP7080 promotes HCC cells proliferation, migration and invasion by regulating miR-601/SIRT1 signal axis. Hence, our finding reveals that lncRNA PP7080 miR-601 may be a novel biomarker and promising therapeutic strategy in HCC pathogenesis.

### High expression of lncRNA PP7080 indicates poor survival prognosis

**Figure 1.**
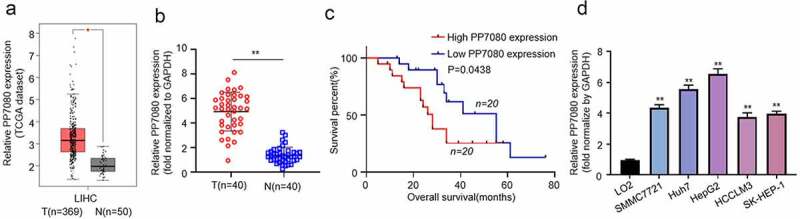
LncRNA PP7080 is highly expressed in HCC and suggests a poor survival prognosis

We analyzed the transcriptome data of HCC cancer tissue (369) and normal tissue (50) in the TCGA database and found that lncRNA PP7080 is highly expressed in HCC. The online analysis results are shown in Figure 1a. Meanwhile, PP7080 expression levels and association with other clinical variables from this validation set are shown in Table 1. QRT-PCR assay was performed to detect the expression of lncRNA PP7080 in 40 pairs of HCC or adjacent tissues. The results showed that lncRNA PP7080 was significantly highly expressed in cancer tissues (Figure 1b). The 40 patients with lncRNA PP7080 expression were divided into groups, the high-expression and low-expression groups were divided by the median value and the survival curve was drawn by KM-plotter. The results suggested that the prognosis of patients with high expression of lncRNA PP7080 is poor (Figure 1c). The expression of lncRNA PP7080 in HCC cell lines (SMMC7721, Huh7, HepG2, HCCLM3 and SK-HEP-1) and the normal liver cell lines (L02) was detected through qRT-PCR, while the expression of lncRNA PP7080 is elevated in HCC cell lines (Figure 1d).

(a) Analysis of the expression of lncRNA PP7080 in liver cancer based on the transcriptome data of HCC cancer tissue (369) and normal tissue (50) in the TCGA database. (b) The expression level of lncRNA PP7080 mRNA detected by RT-PCR in 40 pairs of HCC cancer tissues and adjacent tissues. (c) Survival curve drawn by KM-plotter on the expression of lncRNA PP7080 in 40 patients. (d) The expression level of lncRNA PP7080 mRNA detected by RT-PCR in HCC cell lines (SMMC7721, Huh7, HepG2, HCCLM3 and SK-HEP-1) and the normal liver cell lines (L02). Data were representative of three independent experiments and expressed as mean ± SD (*p < 0.05, **p < 0.01).

**Table 1.** PP7080 expression and clinical characteristics of the HCC patients

Variable	Number	PP7080 expression		*p*-Value
		Low	High	
*Age (years)*				0.68
<60	11	7	4	
≥60	29	15	14	
*Gender*				0.93
Male	22	14	8	
Female	18	10	8	
*Viral hepatitis*				0.95
Type B	37	22	15	
Type B and C	3	2	1	
*Tumor size (cm)*				0.03
<3	10	7	3	
≥3	30	6	24	
*TNM*				0.02
I–II	19	15	4	
III–IV	21	8	13	
*Vascular invasion*				0.02
No	23	17	6	
Yes	17	6	11	
*Lymph nodes metastasis*				0.67
No	22	16	6	
Yes	18	6	12	
*Serum AFP (ng/ml)*				0.93
<25	11	6	5	
≥25	29	17	12	

### Knockdown of lncRNA PP7080 inhibits the proliferation, migration and invasion of HCC cells

**Figure 2.**
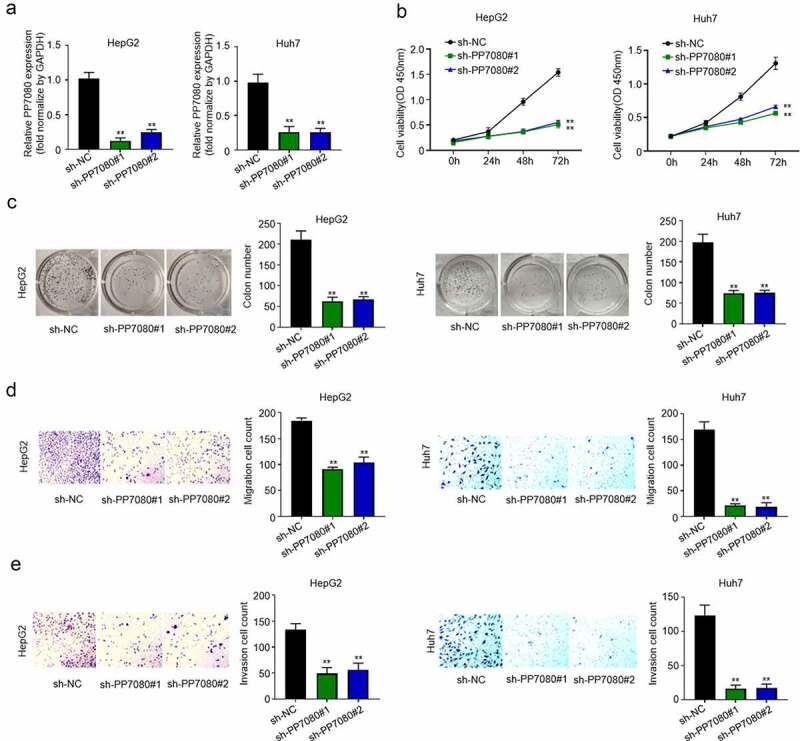
Knockdown of LncRNA PP7080 inhibits the proliferation, migration and invasion of HCC cells

We designed two lncRNA PP7080 interfering RNAs (sh-PP7080#1, sh-PP7080#2) to construct stable knockdown cells by infecting HCC cells Huh7 and HepG2 with lentivirus and detect knockdown efficiency by qRT-PCR. Compared with the control group sh-NC, the two interfering RNAs sh-PP7080#1 and sh-PP7080#2 effectively knock down the expression of lncRNA PP7080 by 50% (Figure 2a). The CCK8 assay showed that lncRNA PP7080 knocking down significantly reduces the growth vigor of Huh7 and HepG2 (Figure 2b). The results of the colony formation experiment show that knocking down PP7080 can effectively inhibit the colony formation ability of HepG2 cells (Figure 2c). As shown in Figure 2d, the transwell experiment was used to detect the effect of HepG2 transfection with sh-PP7080#1 and sh-PP7080#2 on cell migration, while knockdown of lncRNA PP7080 can effectively inhibit the migration ability of HepG2 cells. Then, the transwell experiment was used to detect the effect of HepG2 transfection with sh-PP7080#1 and sh-PP7080#2 on cell invasion. The results indicated that knocking down lncRNA PP7080 can effectively inhibit the invasion ability of HepG2 cells (Figure 2e).

(a) Detection of knockdown efficiency of lncRNA PP7080 by qRT-PCR. (b) Knockdown of lncRNA PP7080 induced HepG2 and Huh7 cell viability detection by CCK8 assay of sh-NC group and sh-PP7080 group. (c) Knockdown of lncRNA PP7080 induced HepG2 colony forming ability detection by colony forming assay of sh-NC group and Sh-PP7080 group. (d) The effect of knockdown of lncRNA PP7080 on HepG2 and Huh7 cell migration detected by transwell experiment. (e) The effect of knockdown of lncRNA PP7080 on HepG2 and Huh7 cell invasion detected by transwell experiment (with Matrigel). Data are representative of three independent experiments and expressed as mean ± SD (**p < 0.01).

### lncRNA PP7080 acts as a molecular sponge for miR-601 in HCC cells

**Figure 3.**
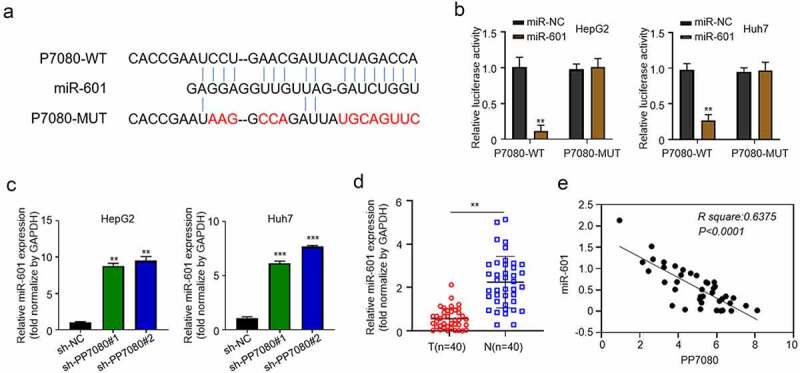
LncRNA PP7080 regulates HCC by targeting miR-601

According to online prediction by DIANA TOOLS software, lncRNA PP7080 has a binding site for targeted adsorption of miR-601. The binding site is shown in Figure 3a, and the mutation sequence of this site is designed. The above binding sites and the mutated sequences were constructed into the luciferin reporter gene, which was transfected into HepG2 respectively, and then overexpressed miR-601 to detect the expression of luciferin. Compared with the control group Mir-NC, the miR-601 overexpression effectively inhibited the expression of luciferin, while the expression of luciferin was not affected when the binding site was mutated (Figure 3b). QRT-PCR detection found that knockdown of lncRNA PP7080 can effectively upregulate the expression of mir-601 in HepG2 (Figure 3c). Among the 40 pairs of HCC and adjacent tissues we collected previously, the expression of miR-601 was significantly lower in cancer tissues (Figure 3d). We further conducted a Pearson correlation analysis on the expression of lncRNA PP7080 and miR-601 in cancer tissues of 40 cancer patients, and found that the expression of miR-601 and lncRNA PP7080 showed a significant negative correlation trend (Figure 3e).

(a) The targeted binding site of lncRNA PP7080 is predicted online by DIANA TOOLS software, the binding site is displayed and the mutation sequence of the site is designed. (b) The above-mentioned binding site and the mutated sequence were constructed into the fluorescein reporter gene, and the expression of fluorescein was detected after HepG2 or Huh7 was transfected with miR-NC or miR-601, respectively. (c) The effect of knockdown of lncRNA PP7080 on the expression of miR-601 was detected by qRT-PCR. (d) The expression levels of miR-601 in 40 pairs of HCC cancer tissues and adjacent tissues were detected by qRT-PCR. (e) The Pearson correlation analysis of the expression of lncRNA PP7080 and miR-601 in the cancer tissues of 40 cancer patients. Data are representative of three independent experiments and expressed as mean ± SD (***p < 0.001).

### miR-601 targets and regulates the expression of SIRT1 gene in HCC

**Figure 4.**
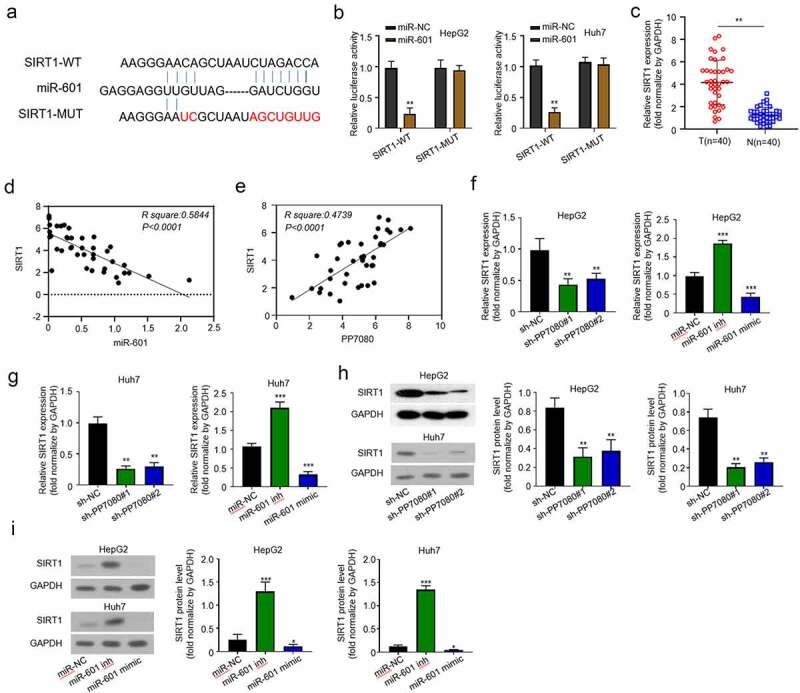
MiR-601 targets and regulates the expression of SIRT1 gene in HCC

Online predictions by Starbase software revealed that miR-601 targeted the 3ʹUTR (400–407) sequence region of SIRT1. We showed the binding site in Figure 4a and designed the mutation sequence for the binding site. The above-mentioned binding site and the mutated sequence were constructed into the fluorescein reporter gene, and then miR-601 was overexpressed to detect the expression of fluorescein. Compared with the control group miR-NC, overexpression of miR-601 effectively inhibited the expression of fluorescein, and when the binding site was mutated, the expression of fluorescein was not affected (Figure 4b). We then performed qRT-PCR assay to analyze SIRT1 mRNA expression level among the 40 pairs of HCC and adjacent tissues collected (Figure 4c). The correlation between the expression of SIRT1 and lncRNA PP7080 or miR-601 in the cancer tissues of 40 cancer patients was analyzed. As shown in Figure 4d, e, the expression of SIRT1 is positively correlated with lncRNA PP7080 and negatively correlated with miR-601. The above results were consistent with the results that knocking down lncRNA PP7080 effectively downregulates the expression of SIRT1 in HepG2 (Figure 4f, g). The above results in vitro and in vivo showed that miR-601 inhibited SIRT1 expression in HCC.

(a) The sequence region of miR-601 targeted to bind SIRT1 was predicted online by Starbase software. The figure shows the binding site and design of the mutation sequence of the site. (b) The above-mentioned binding site and the mutated sequence were constructed into the fluorescein reporter gene, and the expression of fluorescein was detected after HepG2 or Huh7 was transfected with miR-NC or miR-601, respectively. (c) The expression levels of SIRT1 in 40 pairs of HCC cancer tissues and adjacent tissues were detected by qRT-PCR. (d, e) The Pearson correlation analysis of the expression of SIRT1 and lncRNA PP7080 or miR-601 in the cancer tissues of 40 cancer patients. (f, g) The effect of knockdown of lncRNA PP7080 or miR-601 on the expression of SIRT1 was detected by qRT-PCR in HepG2 or Huh7 cells. (g–i) The effect of knockdown of lncRNA PP7080 or miR-601 on the expression of SIRT1 was detected by western blot in HepG2 or Huh7 cells. Data are representative of three independent experiments and expressed as mean ± SD (**p < 0.01; ***p < 0.001).

### LncRNA PP7080 regulates HCC by targeting the miR-601/SIRT1 signal axis

**Figure 5.**
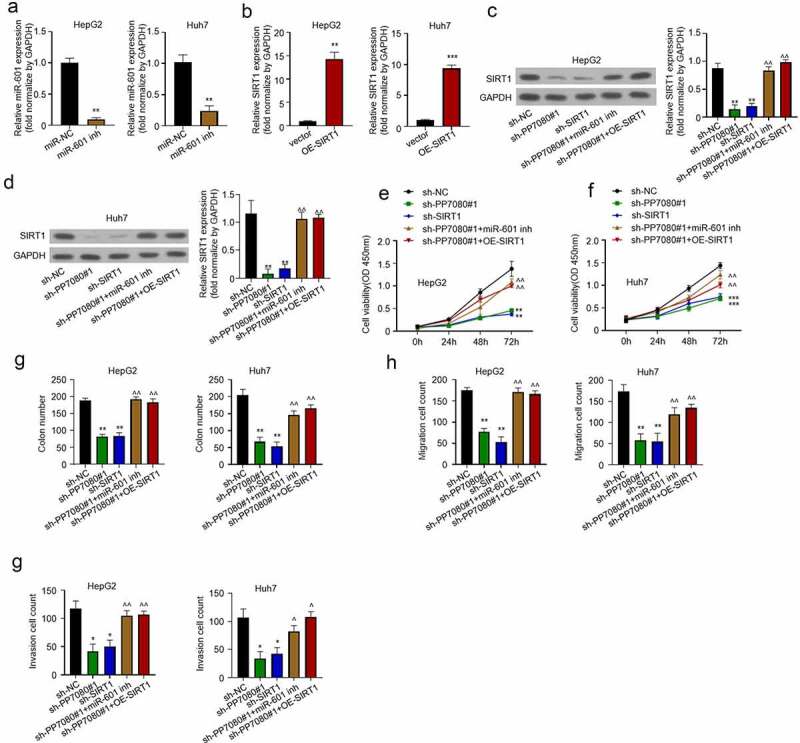
LncRNA PP7080 promotes the malignant progression of HCC through the miR-601/SIRT1 signal axis

The miR-601 inhibitor miR-601inh was synthesized and significantly knocked down the expression of miR-601 compared to miR-NC (Figure 5a). The SIRT1 gene was constructed into an overexpression vector and transfected into HepG2 or Huh7 cells. The overexpression vector OE-SIRT1 significantly increases the expression of SIRT1 (Figure 5b). The expression of SIRT1 in HepG2 cells co-transfected with miR-601inh or OE-SIRT1 was detected by WB. Figure 5c shows that lncRNA PP7080 knockdown significantly reduced the expression of SIRT1. The results showed that knocking down lncRNA PP7080 significantly reduced the cell viability of HepG2 (Figure 5d). The migration ability of Huh7 and HepG2 co-transfected with miR-601inh or OE-SIRT1 was detected by transwell experiment. We found that lncRNA PP7080 knockdown significantly reduced the migration of HepG2 (Figure 5e). Then the transwell experiment (with Matrigel) was used to detect the invasion ability of HepG2 that knocked down lncRNA PP7080 and co-transfected miR-601inh or OE-SIRT1, which showed that knockdown of lncRNA PP7080 significantly alleviated the invasion ability of HepG2 cells (figure 5f).

(a) The inhibition efficiency of miR-601inh on mir-601 was detected by qRT-PCR. (b) Transfection efficiency test of SIRT1 overexpression in HepG2 or Huh7, the expression level of SIRT1 mRNA was detected by qRT-PCR. (c, d) Detection of the expression of SIRT1 protein in HepG2 or Huh7 after cells were co-transfected with miR-601inh or OE-SIRT1 after lncRNA PP7080 knockdown by WB of sh-NC group, sh-PP7080#1 group, sh-PP7080#1+ miR-601inh group and sh-PP7080#1+ OE-SIRT1 group. (e, f) Co-transfected with miR-601inh or OE-SIRT1 after lncRNA PP7080 knockdown induced HepG2 or Huh7 cell viability detection by CCK8 assay of sh-NC group, sh-PP7080#1 group, sh-PP7080#1+ miR-601inh group and sh-PP7080#1+ OE-SIRT1 group. (g) The effect of co-transfected with miR-601inh or OE-SIRT1 after lncRNA PP7080 knockdown on Huh7 and HepG2 cell colony forming detected by transwell experiment. (h) The effect of co-transfected with miR-601inh or OE-SIRT1 after lncRNA PP7080 knockdown on Huh7 and HepG2 cell migration detected by transwell experiment. (i) The effect of co-transfected with miR-601inh or OE-SIRT1 after lncRNA PP7080 knockdown on Huh7 and HepG2 cell invasion detected by transwell experiment (with Matrigel). Data are representative of three independent experiments and expressed as mean ± SD (**p < 0.01; ***p< 0.001).

The above data showed that lncRNA PP7080 is highly expressed in HCC and indicates poor prognosis. We found that lncRNA PP7080 promotes the malignant process of HCC may be regulated by targeting the mir-601/SIRT1 signal axis. In the future, whether lncRNA PP7080 could provide a reference for the diagnosis and treatment of HCC still needs more and more in-depth research support.

## Discussion

Recently, accumulating studies revealed that the abnormal expression of noncoding RNAs such as miRNA and lncRNA, acting as an oncogene or tumor suppressor gene is involved in various human cancers and related to tumor occurrence and poor prognosis [3]. It has been reported that lncRNA PP7080, a novel oncogenic lncRNA, was aberrant expression in colon adenocarcinoma (COAD), and it keeps promising as a diagnostic and prognostic biomarker for COAD [14]. However, the role of lncRNA PP7080 in HCC progression remains unclear. Hence, the present study aims to confirm the relationship between lncRNA PP7080 with HCC and investigate the underlying mechanism of lncRNA PP7080 in HCC progression.

Although previous study demonstrated that lncRNA PP7080 level exhibits high expression in COAD, its biological functions remain unknown. Interestingly, we confirmed that lncRNA PP7080 level was increased in HCC tissues compared to normal adjacent tissues after analyzing the expression data of lncRNA PP7080 in the public database TCGA. The expression of lncRNA PP7080 was related with grade, advanced and volume of HCC, and the elevated expression of lncRNA PP7080 usually indicated poor clinical prognosis of HCC patients.

Generally, one of the functional modes of lncRNAs is as a ceRNA, which influences the functional role of miRNAs through sequence complementation or spongy miRNAs [3]. To investigate the underlying mechanism of lncRNA PP7080 in HCC progression, the potential targets of lncRNA PP7080 were firstly predicted through the software DIANA TOOLS. Fortunately, we found that lncRNA PP7080 could directly target miR-601 and that lncRNA PP7080 silencing significantly upregulated the miR-601 level. Moreover, the expression of lncRNA PP7080 and miR-601 was also negatively correlated in the HCC tissue of our cohort. Notably, miR-601 is underexpressed in many kinds of tumors and has a suppressive effect on proliferation, migration and invasion in carcinomas as a tumor suppressor gene [19–21]. Additionally, it was reported that the expression of miR-601 exhibited low level in prostate cancer stem cells, and miR-601 also inhibited the proliferation of pancreatic cancer cells by targeting and regulating KRT5 [22]. Herein, our findings indicated that the restorative effect of miR-601 on cell proliferation, migration, invasion and apoptosis is triggered by lncRNA PP7080 knockdown, suggesting that lncRNA PP7080 acts as a molecular sponge for miR-601 and lncRNA PP7080/miR-601 axis may be essential elements for HCC progression.

Recent reports show that the expression of SIRT1 protein is significantly overexpressed in non-tumor liver tissues adjacent to cancer, and the elevated SIRT1 level is related to tumor grade and predicts poor prognosis [23,24]. Interestingly, we confirmed that SIRT1 is the target molecule of miR-601 in HCC cells in this study. Additionally, previous study revealed that overexpression of SIRT1 reversed the function of miR-601 on pancreatic cancer cells [25]. Intriguingly, our results indicated that lncRNA PP7080 was positively correlated with SIRT1, and miR-601 is negatively correlated with SIRT1. Importantly, lncRNA PP7080 knockdown significantly suppressed SIRT1 expression and further inhibited the proliferation, colony formation, migration and invasion of HCC cells, whereas miR-601 downregulation could reverse such effect. Previous study reported that the inhibition of HCC cell proliferation and clone formation might be related to cell senescence [26]. Therefore, whether SIRT1 causes the senescence of HCC cells is worth further investigation. In summary, our study indicated that lncRNA PP7080 has oncogenic effects and it could promote HCC proliferation, migration and invasion via modulation of miR-601/SIRT1 axis.

## Conclusion

In summary, our research provides a novel view that the lncRNA PP7080/miR-601/SIRT1 axis is involved in the growth of HCC, which indicated that lncRNA PP7080/miR-601/SIRT1 axis may be a potential diagnosis and therapeutic biomarker of HCC.

## Supplementary Material

Supplemental Material

## Abbreviations

FBS: fatal bovine serun, BSA: Bovine Serum Albumin, TBST: TBST buffer, PBS: phosphate buffer saline, TNM: Tumor Node Metastasis, AFP: alpha fetoprotein, WB: western blot.

## Highlights

1. LncRNA PP7080 was highly expressed in HCC and predicted a poor prognosis.
2. LncRNA PP7080 directly target miR-601 as a molecular sponge.
3. LncRNA PP7080 promoted HCC cells proliferation, migration and invasion by downregulate miR601/SIRT1 signal axis.
4. LncRNA PP7080 knockdown led to inhibition of HCC progression.
