# Supplementary figures and images for: Long noncoding RNA PP7080 promotes hepatocellular carcinoma development by sponging mir-601 and targeting SIRT1
Source: Bioengineered. 2021 May 6;12(1):1599–610. doi: 10.1080/21655979.2021.1920323 (PMC8806262; doi:10.1080/21655979.2021.1920323)

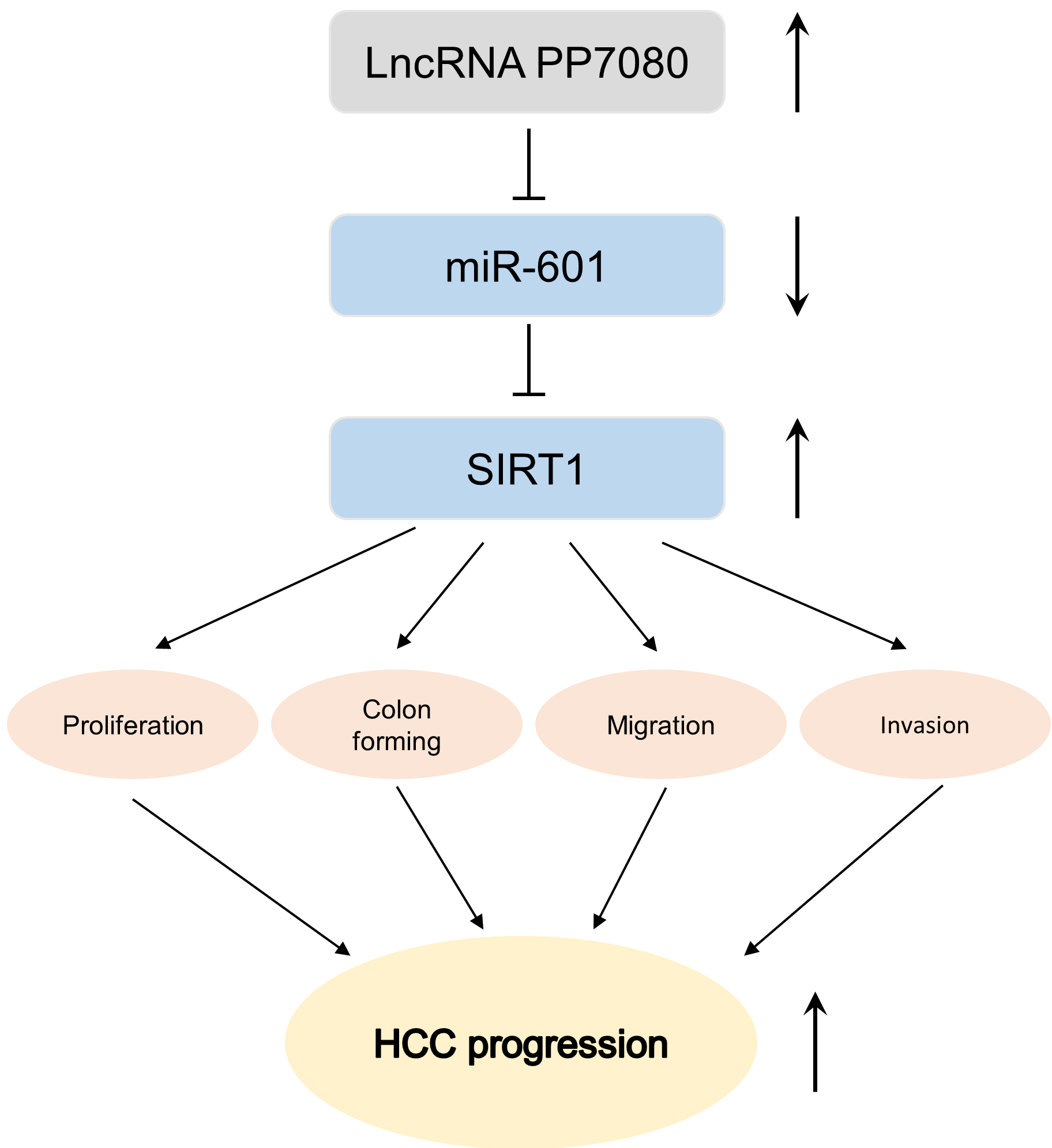

Supplement: Supplemental Material [file KBIE_A_1920323_SM1783.tif]
